# Study on the Interactions Between Caffeoylquinic Acids With Bovine Serum Albumin: Spectroscopy, Antioxidant Activity, LC-MS^n^, and Molecular Docking Approach

**DOI:** 10.3389/fchem.2019.00840

**Published:** 2019-12-06

**Authors:** Qishu Jiao, Wei Zhang, Yanyan Jiang, Lijuan Jiang, Xiangyang Chen, Bin Liu

**Affiliations:** School of Chinese Materia Medica, Beijing University of Chinese Medicine, Beijing, China

**Keywords:** caffeoylquinic acids, bovine serum albumin, spectroscopy, docking simulation, HPLC-MS/MS, antioxidant activity

## Abstract

Clarified the binding mechanism of drugs with plasma proteins could provide fresh insights into the drug development. Caffeoylquinic acids (CQAs) are a kind of phenolic acid compounds which has extensive biological effects. This study investigated the binding mechanism of three CQAs, including chlorogenic acid, neochlorogenic acid, and cryptochlorogenic acid, with bovine serum albumin (BSA) by using multi-spectroscopic techniques, including fluorescence, UV-Vis, Fourier transform infrared (FT-IR) and circular dichroism (CD) spectroscopy, LC-MS^n^, molecular docking and antioxidant activity assessment. In addition, the influences of PBS buffer, Tris-HCl buffer and water as solvents on the characteristics of CQAs and BSA interaction were also investigated. The results showed that intrinsic fluorescence of BSA was quenched by CQAs and the interaction was static quenching with the formation of a non-fluorescent complex. The binding of CQAs and BSA was spontaneous, and Van der Waals forces and hydrogen-bond interaction occupied crucial roles in the binding. All the three CQAs could bind to Site I in Domain IIA. The weakest interaction between neochlorogenic acid and BSA may due to its larger polarity. The results also indicated that the binding affinity of CQAs had a descending order of Tris-HCl > H_2_O > PBS. This study firstly clarified the binding mechanism of CQAs with BSA and changes of the binding in different solvents, and provided fresh insights into this drug transportation and metabolism.

## Introduction

**Figure 1:**
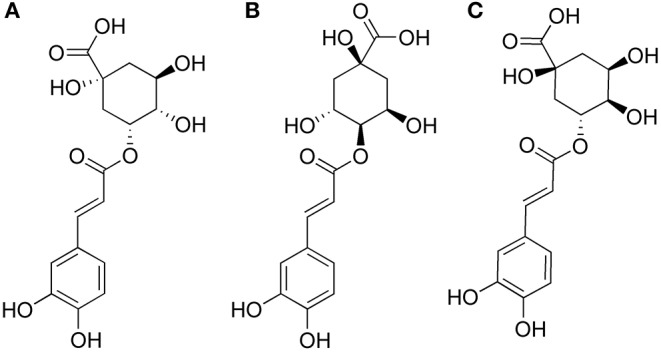
The chemical structures of 3-CQA **(A)**, 4-CQA **(B)**, and 5-CQA **(C)**.

Phenolic acid compounds are a series of dietary antioxidants which are belong to secondary metabolism of many plants. Due to their multiple active phenolic groups, phenolic acid compounds have good antioxidant, anti-microbial and anti-cancerogenic effects (Cui et al., 2002; Kacem et al., 2015; Zhao et al., 2015). Among them, chorogenic acids (CGAs), as internationally acknowledged plants gold, are the most common phenols which are widespread exist in coffee, strawberries, pineapples, apples, sunflowers, and blueberries. The vast majority of CGAs were found belong to three groups: caffeoylquinic acids (CQAs), dicaffeoylquinic acids (di-CQAs), and feruloylquinic acids (FQA) (Clifford et al., 2006). CQAs are a series of esters formed between one molecule of caffeic acid and quinic acid, such as chlorogenic acid (5-*O*-caffeoylquinic acid, 5-CQA), neochlorogenic acid (3-*O*-caffeoylquinic acid, 3-CQA), and cryptochlorogenic acid (4-*O*-caffeoylquinic acid, 4-CQA). The chemical structures of 3-CQA, 4-CQA and 5-CQA are shown in Figure 1. As the only CGA commercially available, 5-CQA has multiple functions such as anti-oxidative, anti-bacterial, anti-inflammatory, anti-hypertensive, cardiovascular protection and regulation of glycolipid metabolism activities (Upadhyay and Mohan Rao, 2013). As two monoacyl isomers of 5-CQA, 3-CQA and 4-CQA show the highly similar chemical structures and strong anti-oxidative activity like 5-CQA (Bajko et al., 2016).

The binding of plasma protein and drugs was a usual and reversible process when drugs entered into the bloodstream (Vuiginer et al., 2012). Human serum albumin (HSA) is the most abundant protein in plasma, which could bind many endogenous compounds, such as amino acid, fatty acid, bile acid, hormone, toxic metabolite, and drug. The binding degree of small molecules and HSA will directly influence the concentration of the free small molecules, and then influence the absorption, distribution, metabolism, excretion, and toxicity (ADME/T) (Gonciarz et al., 2012). Therefore, the investigations of HSA binding with small molecules are of important significance. Bovine serum albumin (BSA) was the most common substitution model for HSA due to its highly similar tertiary structure (76.52%) (Gelamo and Tabak, 2000; Jiao et al., 2018).

For the past few years, extensive studies concentrated on the binding of CGAs with proteins. Li et al. found that the binding affinities were decreased when substituted of methoxyl group for caffeic acid, while, the CGA which was the esterification production of caffeic acid with quinic acid had the weaker protein binding capacity (Li et al., 2010). Bin Tang et al. thought that the binding capability of CGAs increased with the increased caffeoyl group and the substituted positions of caffeoyl group and the formation of ester bond in CQAs also could moderately effect the binding process (Tang et al., 2016). The binding of drug and protein is influenced by multiple factors, such as the pH, ionic concentration of solvents, temperatures, trace elements, and solvent types (Jiao et al., 2018). In generally, to accurately simulate the human body environment, PBS and Tris-HCl buffer are two most commonly used buffers in drug-protein binding experiment to keep the pH of the BSA solution at 7.40. However, due to the presence of ester bond, unsaturated bond and phenolic hydroxyl groups, CQAs are always unstable in neutral or alkaline solution, and hydrolytic decomposition or lactone transfer are often happened (Trugo and Macrae, 1984). Therefore, different buffer solution could influence the stability of CQAs, and then change the binding affinity with BSA.

In this study, the interactions of 3-CQA, 4-CQA, and 5-CQA with serum albumin were explored by multi-spectroscopic methods, molecular docking and antioxidant activity assessment. In addition, the influences of PBS buffer, Tris-HCl buffer and water (H_2_O) as solvents on the characteristics of CQAs and BSA interaction were also investigated. Binding parameters were calculated according to fluorescence data. Synchronous fluorescence, 3D fluorescence, UV-Vis, Fourier transform infrared (FT-IR) and circular dichroism (CD) spectroscopy were used to determine protein conformational changes influenced by drugs. Molecular docking and antioxidant activity assessment were also conducted to further certify the interactions. Eventually, an HPLC-Q-TOF MS^n^ method was employed to detect the degradation behavior of CQAs in BSA solution, which could explain the binding affinity differences. This study not only could deepen the understanding of the binding mechanisms of CQAs with BSA, but also give some worthwhile information for the design and pharmaceutical research of new drugs.

## Materials and Methods

### Materials

BSA was purchased from AMRESCO. Chlorogenic acid, cryptochlorogenic acid and neochlorogenic acid were all purchased from Chengdu Must Bio-Technology Company in China. Ibuprofen and Warfarin Sodium were purchased from Shanghai Yuanye Bio-Technology Company in China. Digoxin was purchased from Tokyo Chemical Industry Company in Japan. BSA solution was prepared to 1.0 × 10^−4^ M with water. PBS and Tris-HCl buffer were prepared to 5.0 × 10^−2^ M, all the solution were kept at pH 7.4 and contain 0.1 M NaCl. The stock solution of 3-CQA, 4-CQA, 5-CQA, ibuprofen, warfarin sodium, and digoxin were directly dissolved in MeOH as stock solution (1.0 × 10^−3^ M).

### Fluorescence Spectrum Measurements

Fluorescence analysis was determined with the LS-45 fluorophotometer (PerkinElmer, USA). The concentration of BSA was set as 0.2 × 10^−6^ M, while the 3-CQA, 4-CQA, 5-CQA concentrations were 0.0, 1.0, 1.5, 2.0, 2.5, 3.0, 3.5, 4.0, 4.5, 5.0 × 10^−6^ M. Before the fluorescence measurement, the drug-BSA solution was vortex-mixed for 2 min and incubated for 15 min at 27°C (300 K), 32°C (305 K), and 37°C (310 K). The excitation wavelength and emission wavelength were, respectively, set as 280 nm and 290–500 nm. The synchronous fluorescence spectrum was recorded at Δλ = 15/60 nm at 300 K. The concentration of BSA was set as 0.2 × 10^−6^ M, and the concentrations of CQAs were set as 0~5.0 × 10^−6^ M by successive additions. The 3D fluorescence spectrum of BSA (0.2 × 10^−6^ M) and the BSA-drug (10:1 mol ratio of protein to ligand) complexes were determined at 200–350 nm in 5 nm increments, and the emission spectrum was set as between 200 and 500 nm.

### Site Marker Competitive Experiment

The binding sites of CQAs with BSA were studied by a fluorescence titration method. Warfarin sodium, ibuprofen, and digoxin were chosen as the marker of site I, site II, and site III of BSA, respectively. The concentrations of BSA and CQAs were kept at 0.2 × 10^−6^ and 2.0 × 10^−6^ M, respectively. Various concentrations of probes solution were gradually added to the CQAs-BSA complex. The emission wavelength and excitation were, respectively set as 290–500 nm and 280 nm.

### UV-Vis Absorption Measurements

Absorbance measurement was carried out an UH5300 UV-Vis spectrophotometer (Hitachi, Japan), recording from 200 to 400 nm. The BSA solution was used with a constant concentration of 0.2 × 10^−6^ M and CQAs concentration varied from 0.2 × 10^−6^ M to 0.6 × 10^−6^ M. The absorption spectra were obtained at 300 K.

### FTIR Measurement

FTIR spectrum of BSA and CQAs-BSA was detected on a Nexus 470 FTIR spectrometer (USA) equipped with the attenuated total reflection (ATR) accessory. FTIR spectra were taken by the ATR method with the wave range from 400 to 4,000 cm^−1^ with 60 scans and resolution was set as 4 cm^−1^. The spectra of BSA (2 × 10^−5^ M) and CQAs-BSA complexes (1:5 mol ratio of protein to ligand) were all recorded at room temperature. Meanwhile, the FTIR spectrum of PBS buffer, Tris-HCl buffer, sodium chloride solution and free CQAs solutions was also determined under the same conditions. The free BSA spectrum was obtained by the spectrum of BSA in buffer subtracted with that of the buffer. The FTIR spectrum of CQAs-BSA complex was gained via the spectrum difference of BSA and CQAs solution in corresponding solvents.

### CD Measurement

The J-815 automatic recording spectropolarimeter (Japan) equipped with 1.0 mm path length quartz cuvettes was employed to determine the CD spectrum of BSA and CQAs-BSA. All the CD spectra were recorded from 190 to 250 nm at 300 K, and the scanning speeds were all set as 50 nm·min^−1^. The concentration of BSA and CQAs was 2.0 × 10^−6^ and 10.0 × 10^−6^ M, respectively. Each spectrum was scanned for successive three times and corrected by corresponding buffer solution.

### Measurements of Antioxidant Capability

The determination of FRAP was accomplished by T-AOC Assay Kit (Beyotime). Stock solution contained TPTZ diluent, TPTZ solution, detection buffer, FeSO_4_·7H_2_O and Trolox solutions. FRAP working solutions were disposed by TPTZ diluent, TPTZ solution and detection buffer with the ration of 150:15:15. FeSO_4_ solution (100 mM) was diluted to 0.15, 0.30, 0.60, 0.90, 1.20, and 1.50 mM, which was used to make standard curve. The concentration of BSA and CQAs was 2.50 × 10^−5^ M and 5.00 × 10^−4^ M, respectively. Five microliter of samples or FeSO_4_ solutions with various concentrations were mixed with 180 μL of FRAP working solutions for 5 min at 37°C. The record wavelength of the reaction mixture was then set as 593 nm. The total antioxidant capacities of samples were reckoned by standard curve and expressed by the concentration of FeSO_4_.

ATBS was assayed by T-AOC Assay Kit (S0121, Beyotime, Shanghai, China). Stock solutions included detection buffer, ATBS solutions, hydrogen peroxide solutions, catalases, and Trolox solutions. The hydrogen peroxide solutions and catalase were diluted by a factor of 1,000 and 10 with ultrapure water and detection buffer, respectively. ATBS working solutions were prepared by detection buffer, ATBS solutions and diluted hydrogen peroxide solution with the ration of 152:10:8. Trolox solution (10 mM) was diluted to 0.15, 0.30, 0.60, 0.90, 1.20, and 1.50 mM, which was used to make standard curve. The concentration of samples was same as FRAP method. Twenty microliter of diluted catalase, 10 μL of samples or Trolox solutions with various concentrations and 170 μL of ATBS solution were sequentially placed in 96-well-plates and keep at 25°C for 6 min. The detection wavelength was set as 414 nm. The total antioxidant capacity of samples were calculated by standard curve and expressed by the concentration of Trolox. The total antioxidant capacity of CQAs before interaction with BSA could be expressed in the difference value of which of CQAs with buffer solution. The total antioxidant capacity of CQAs after interaction with BSA was shown by the difference value of which of drug-BSA complex and BSA.

### HPLC-MS/MS Analysis

HPLC coupled with Agilent 6520 Q-TOF mass spectrometers with ESI sources was employed in this study. All the samples were analyzed on a Waters Sunfire C_18_ column at 25°C. The mobile phase was composed of acetonitrile (A) and 0.1% (*v/v*) formic acid-water (B). The gradient program was as follows: 0–5 min, 5–10% B; 5–10 min, 10% B, 10–15 min, 10–20% B; 20–30 min, 20–90% B. The corresponding parameter of mass was set as follows: temperature was 300°C; flow rate was 10.0 L·min^−1^; nebulizer gas with a pressure of 30 psig; fragmentor with a voltage of 120 V; and capillary with a voltage of 3,500 V. The mass ranges for the MS scan were record from *m/z* 100 to 1,000, and the mass ranges of MS^2^ were set from *m/z* 50 to 1,000. Agilent LC-Q-TOF-MS Mass Hunter Acquisition Software Version A.01.05 was employed to operate, acquire, and analyze the data. The data acquisition was carried out by Mass Hunter Acquisition Software Version B.02.00.

A certain volume of 5-CQA solutions were vortex mixed with BSA solutions for 30 s, and the mixtures were keep at 27°C for 12 h. The concentration of 5-CQA and BSA were all 1 mM. After reaction finished, 200 μL of the mixture were vortex mixed with 600 μL of methanol for 30 s and centrifuge at 8 000 rpm for 5 min. Supernatants (500 μL) were collected and dried under high purity nitrogen at 25°C. Two hundred microliter methanol-water (1:1) was used to re-dissolved the residues, vortex mixed for 30 s and then centrifuged at 8,000 rpm for 5 min. The supernatants were injected into HPLC-Q-TOF-MS^n^ spectrometer system for analysis.

### Molecular Docking

The 3D structure of CQAs and their degradation products directly downloaded in sdf format from the PubChem database (https://pubchem.ncbi.nlm.nih.gov). The crystal structures of the BSA (PDB ID: 4OR0) were obtained from the Protein Data Bank database (https://www.rcsb.org). The protein was disposed by deleting B chain, removing co-crystallized water, adding polar hydrogen atoms and minimizing energy. Original ligand was extracted and re-docked into the corresponding active pocket where the original ligand located. Libdock module in Discovery Studio 4.0 was performed to conduct docking simulation. The root-mean-square deviation (RMSD) between re-docking pose and original ligand conformation was calculated to evaluate docking reliability and the applicability of the protein model. The binding efficiency of each target to the original ligand and prototype compounds was measured using LibDock score. LibDock score of original ligand was set to the threshold to screen the potential compound interacting with target.

## Results and Discussion

### Fluorescence Spectrum

**Figure 2:**
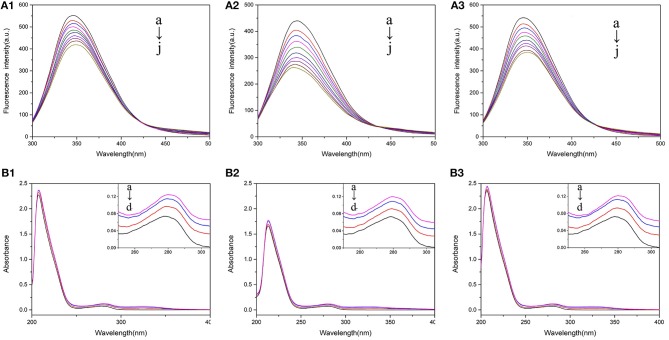
**(A)** The fluorescence spectra of 2.0 × 10^−7^ M BSA mixed with various concentrations of 5-CQA at 300 K in PBS buffer (1), Tris-HCl buffer (2), and water (3). The concentration of 5CQA is 0.0, 1.0, 1.5, 2.0, 2.5, 3.0, 3.5, 4.0, 4.5, 5.0 × 10^−6^ M from a to j. **(B)** UV-Vis absorption spectra of 2.0 × 10^−7^ M BSA in the presence of 3-CQA **(A)** and 4-CQA **(B)** in PBS buffer (1), Tris-HCl buffer (2), and H_2_O (3). The concentration of 3-CQA and 4-CQA is 0, 2, 4, 6 × 10^−6^ M from a to d.

Fluorescence spectrum could reflect the information of tyrosine (*Tyr*) and tryptophan (*Trp*) when the excitation wavelength was set as 280 nm (Calce et al., 2013). Figure 2A showed the fluorescence quenching of BSA with 5-CQA, and that of BSA with 3-CQA and 4-CQA was shown in Supplementary Figure 1. As the increase of concentration of CQAs, the fluorescence intensities of BSA were all decrease. CQAs showed the highest fluorescence quenching rate in Tris-HCl buffer, indicating their stronger binding abilities to BSA in this solution. The influences of CQAs to the fluorescence spectra of BSA were shown in Supplementary Table 1. For CQAs-BSA system, the red shifts could be observed in PBS buffer and H_2_O, but blue shifts happened in Tris-HCl buffer. These changes reflected that all the three CQAs could interact with BSA and change the microenvironment of BSA. The microenvironment of *Tyr* and *Trp* residues was more hydrophobic after CQAs interaction with BSA in Tris-HCl buffer solution, which could be the main reason of the strongest BSA affinity.

### Mechanisms of Fluorescence Quenching

Static and dynamic quenching were two kinds of quenching mechanisms. Both of them followed the equation of Stern-Volmer (Tang and Jia, 2013):

(1) $$\begin{array}{r} F_{0}/F=1+K_{\text{SV}}\left[Q\right] \end{array}$$

where *F*_0_ and *F* were the fluorescence intensities of BSA with or without CQAs, respectively; *K*_SV_ were the Stern-Volmer quenching constants; [*Q*] was the concentration of CQAs.

Generally speaking, the mechanism of static quenching was a new no-fluorescence complex formatted. The collision of excited-state fluorescent derivatives with the quencher was the main reason for dynamic quenching. Therefore, the temperature rises could decrease the static quenching constant because of the lower stability of drug-BSA complex, while could increase the dynamic quenching constant due to the increased possibility of diffusivity of the molecules and molecular collision. Stern-Volmer plots were shown in Supplementary Figure 2. The *K*_SV_ of 5-CQA-BSA at different temperatures were shown in Table 1, the values of *K*_SV_ of 3-CQA and 4-CQA were listed in Supplementary Tables 2, 3. The results showed that the *K*_SV_ of CQAs was all reduced with increasing temperature, indicating that quenching modes were all static quenching. The decreasing-trend of *K*_SV_ was consistent in different solvent systems. But the values of *K*_SV_ were the biggest in Tris-HCl buffer, which indicated that the strongest binding was happened in Tris-HCl buffer. In conclusion, the interaction of CQAs with BSA all occurred by the static quenching, which was same in the three solvent systems.

**Table 1:** Binding and thermodynamic parameters for the interaction between BSA and 5-CQA at different temperatures.

**Solution**	**T (K)**	***K*_SV_ (L·mol^−1^)**	***K*a (L·mol^−1^)**	***n***	**Δ*H* (kJ·mol^−1^)**	**Δ*S* (J·mol^−1^·k^−1^)**	**Δ*G* (kJ·mol^−1^)**
PBS	300	13.15 × 10^4^	1.47 × 10^6^	1.2198	−210.04	−581.80	−35.50
	305	9.97 × 10^4^	1.51 × 10^5^	1.0374			−32.59
	310	9.65 × 10^4^	0.97 × 10^5^	0.9251			−29.68
Tris-HCl	300	19.70 × 10^4^	3.41 × 10^7^	1.4222	−275.18	−775.31	−42.59
	305	10.95 × 10^4^	2.47 × 10^6^	1.2527			−38.71
	310	6.93 × 10^4^	0.97 × 10^6^	1.2177			−34.83
H_2_O	300	9.03 × 10^4^	5.83 × 10^6^	1.3385	−212.37	−580.03	−38.36
	305	6.94 × 10^4^	7.88 × 10^5^	1.2036			−35.46
	310	5.98 × 10^4^	3.78 × 10^5^	1.1507			−32.56

### Binding Constants and Binding Sites

Binding constants and binding sites could be determined by double logarithmic curve equation (Hemmateenejad et al., 2012):

(2) $$\begin{array}{r} \log\frac{F_{0}-F}{F}=\log K_{a}+n\log\left[Q\right] \end{array}$$

where *F*_0_ and *F* were the fluorescence intensities of BSA with or without CQAs, respectively; [*Q*] was the concentrations of CQAs; *K*_a_ was the binding constants and *n* was the number of binding sites.

The typical plot obtained at 300 K of CQAs in PBS buffer was shown in Supplementary Figure 3. Corresponding *K*_a_ and *n* of the interaction between 5-CQA and BSA in three solvent systems were shown in Table 1, the binding information of 3-CQA and 4-CQA were listed in Supplementary Tables 2, 3. For all the three CQAs, the reduced *K*_a_ with growing temperature indicated the stability of CQAs-BSA reduced as the temperature rose and the interaction of all of CQAs with BSA occurred by the static quenching, which was consistent with quenching constants. Besides that, the binding constants of all the CQAs and BSA in three solvent systems were in 10^5^ magnitudes, which showed that CQAs-BSA complex exhibited a comparatively intense combination. The number of binding sites of all the three CQAs was about 1, which indicated that the complex was composed of BSA and CQAs with a ratio of 1:1 in three solvent systems.

However, the results showed that the affinities of CQAs with BSA were influenced by their molecular structures and solvent environments. In PBS buffer, the order of *K*_a_ of CQAs was 4-CQA (5.70 × 10^6^ M) > 5-CQA (1.47 × 10^6^ M) ≈ 3-CQA (1.41 × 10^6^ M) in 300 K, while the order of which in Tris-HCl buffer and H_2_O were 5-CQA (3.41 × 10^7^ M) ≈ 4-CQA (3.32 × 10^7^ M) > 3CQA (9.27×10^6^ M) and 5-CQA (5.83 × 10^6^ M) ≈ 4-CQA (5.78 × 10^6^ M) > 3-CQA (3.08 × 10^6^ M), respectively. In short, for the CQAs-BSA system, 5-CQA and 4-CQA had larger *K*_a_ than 3-CQA, indicating that 5-CQA and 4-CQA had a much higher affinity than 3-CQA in binding with BSA. The crystal structure of BSA showed that BSA was composed of three homologous domains (I–III) and each domain had two subdomains. Each domain is constituted by a hydrophobic cylinder formed by six helices, and the binding sites of a majority of small molecules are in the hydrophobic-cavum of domain IIA and IIIA. Thus, the serum albumin is more likely binding with weak polarity molecules (Gao et al., 2010). The polarity of three CQAs was in a descending order of 3-CQA > 5-CQA > 4-CQA. In addition, their chromatographic behavior also proved that 3-CQA had much higher polarity than 5-CQA and 4-CQA. Therefore, the lower *K*_a_ of 3-CQA compared with 5-CQA and 4-CQA might due to its larger polarity. Meanwhile, for the solvent environments of Tris-HCl buffer and H_2_O, the binding constants of the three CQAs had the similar rules to those in PBS buffer.

For different CQAs, they had the same variation tendency of *K*_a_ in different solvent systems with a descending order of Tris-HCl > H_2_O > PBS. When the temperature rise from 300 to 310 K, the binding affinity changed of 5-CQA for BSA in three solvent environments was in an order of Tris-HCl (35.2) > H_2_O (15.4) > PBS (15.2). In addition, the affinities of 4-CQA and 3-CQA increased the biggest times of 37.4 and 8.5 in Tris-HCl buffer, respectively. The study of Taha and Lee showed that Tris buffer could stabilize the BSA molecules and the compactness of BSA could be increased (Taha and Lee, 2010). Therefore, it could be the reason for the BSA-CQAs complex could be more stable and the binding constants were higher in Tris buffer.

### Binding Parameters and the Kind of Binding Force

The binding force between drugs and BSA was non-covalent interaction, like hydrogen-bond interaction, electrostatic forces, hydrophobic effects and Van der Waals forces (Cheng et al., 2015). The binding parameters could be employed to determine the types of binding forces. The value of Δ*H* (enthalpy change) and Δ*S* (entropy changes) could be determined by Van't Hoff equation:

(3) $$\begin{array}{r} \ln\;K_{a}=-\frac{\Delta H}{RT}+\frac{\Delta S}{R} \end{array}$$

where *K*_a_ were the binding constants, R were the gas constants and T was the temperatures. Free energy changes (Δ*G*) could be determined by the following equation:

(4) $$\begin{array}{r} \Delta G=\Delta H-T\Delta S=-RT\ln\;K_{a} \end{array}$$

The results of the interaction between 5-CQA and BSA in three solvent systems were shown in Table 1, the values of 3-CQA and 4-CQA were listed in Supplementary Tables 2, 3. Ross et al. found that the binding forces could be distinguished by the magnitude and positive or negative sign of the thermodynamic parameter (Ross and Subramanian, 1981). The values of Δ*H* and Δ*S* of CQAs in three solvent environments were all negative, which indicated that hydrogen-bond interaction and Van der Waals forces were the crucial binding forces. However, Tang et al. thought that because of the easily ionized properties of CQAs, the electrostatic interactions cannot be ignored in every polyphenol-protein interaction (Tang et al., 2016). The negative values of Δ*G* indicated that a spontaneous interaction process between CQAs with BSA was happened. The position change of caffeoyl group and solvent environments did not change the binding forces of CQAs-BSA. However, the absolute values of Δ*G* were different, which determined the interaction intensity of drug-protein binding. For the three CQAs, the absolute values of Δ*G* were found in a descending order of Tris-HCl > H_2_O > PBS. Moreover, the absolute value of Δ*G* of 3-CQA was always the lowest compared with 5-CQA and 4-CQA, which indicated the weakest binding affinity of 3-CQA with BSA. All the results coincided with the binding constants.

### Site Marker Competitive Experiment

There were three binding sites in BSA, which named as Site I, II, and III. Phenylbutazone and warfarin were binding to drug Site I. Ibuprofen and thyroxine were the probe to Site II. Besides, digoxin could bind to Site III (Caruso et al., 2012; Li et al., 2016). Therefore, warfarin, ibuprofen and digoxin were chosen as the fluorescence probes to determine binding sites on protein by drugs. The probe displacement percentage could be used to determine the probe substitution for drugs and could be determined by the following relationship (Gholivand et al., 2013):

(5) $$\begin{array}{r} Displacement\left(\%\right)=\frac{F_{2}}{F_{1}}\times 100\% \end{array}$$

where *F*_1_ and *F*_2_ were the fluorescence intensities with or without drugs, respectively. The results were shown in Supplementary Figure 4. For the three CQAs in PBS buffer, the probe displacement percentage was all decreased with the addition of warfarin, while this was nearly invariable for ibuprofen and digoxin. Therefore, only warfarin and CQAs competed for the same sites, which showed that the binding site of CQAs was Site I in BSA.

### Conformational Changes of BSA Upon Addition of CQAs

#### UV-Vis Spectrum

UV-Vis spectra could reflect the structural changes in drug-protein interactions. Different BSA absorption spectra with various amounts of 5-CQA in different solutions were shown in Figure 2B, and the absorption spectrum of BSA with 3-CQA and 4-CQA were shown in Supplementary Figure 5. As shown in Figure 2B, there were two main absorption peaks of BSA, the stronger peak (208 nm) reflected the peptide chain of BSA, and the weaker peak (280 nm) reflected the aromatic amino acids (Kanakis et al., 2006). As the addition of CQAs, the absorption intensities of BSA at 280 nm reduced, indicating that the binding between the CQAs and BSA changed the conformations of protein. Besides, the absorption spectra of BSA-CQAs showed similar trends in the three solutions, indicating that the solvent environments did not change the interaction modes.

#### FTIR Spectrum

**Figure 3:**
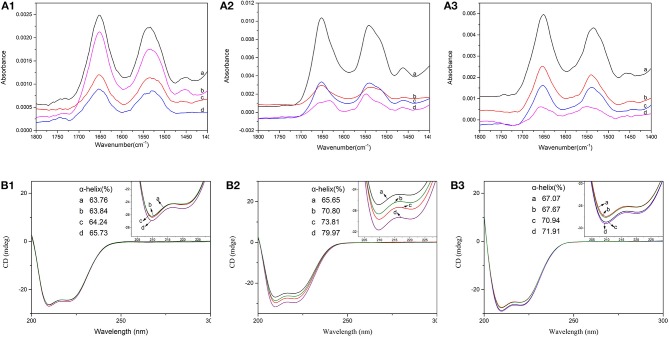
FTIR spectra **(A)** and CD spectra **(B)** of BSA in the absence (a) and presence of 5-CQA (b), 3-CQA (c), and 4-CQA (d) in PBS buffer (1), Tris-HCl buffer (2), and H_2_O (3).

There were eight characteristic absorption peaks of protein on FTIR. Among them, the amide I and amide II band, which, respectively, occurred in 1,600~1,700 cm^−1^ and 1,600~1,500 cm^−1^, could reflect the secondary structure of protein (Lu et al., 2014). The FTIR spectrum of free BSA and BSA-CQAs complex in different solvent environments were shown in Figure 3A. In the FTIR spectra, the absorbance of BSA in Tris-HCl buffer was stronger than that in PBS buffer or H_2_O. Meanwhile, the absorbance changes of BSA-CQAs complex compared with free BSA in Tris-HCl buffer were also the most obvious than that in PBS buffer or H_2_O, and the changes followed the order of Tris-HCl > H_2_O > PBS. The results indicated the secondary structures of BSA were changed due to CQAs in different buffer, and the strongest changes happened in Tris-HCl buffer.

#### CD Spectrum

Far-UV CD spectrum could monitor the secondary structures changes of BSA. To further study the interaction of CQAs and BSA and accurately detect the changes of the secondary structures of BSA, CD spectra of BSA was performed in with or without CQAs in different solvent systems. The CD spectrum of BSA showed two negative peaks at 208 and 222 nm—the characteristic peaks of α-helix structures (Siddiqi et al., 2016). The intensities of CD spectrum could be showed as mean residue ellipticity (MRE), the contents of α-helixes could be determined by the following relationship (Liu et al., 2004):

(6) $$\begin{array}{r} MRE=\theta_{obs}/\left(10nlC_{p}\right) \end{array}$$

(7) $$\begin{array}{r} \alpha -helix\left(\%\right)=\frac{-{\left[\theta \right]}_{208}-4000}{29000}=\frac{-{\left[\theta \right]}_{222}-4000}{33000} \end{array}$$

where *C*_*p*_ were the mole concentrations of the BSA; *n* were the numbers of amino acid residues (583 for BSA) and *l* were the cell path-lengths; [θ]_208_ and [θ]_222_ are the *MRE* values at 208 and 222 nm, respectively.

Figure 3B showed the CD spectrum of BSA with CQAs in different solvents and corresponding α-helix changes. It showed that the intensity of both band at 208 and 222 nm increased with the addition of CQAs with BSA in different solvent environments, but no changes of the position of its peaks were observed. The results showed that α-helix contents of BSA increased for all CQAs-BSA systems in three solvent environments. That could be due to that the hydrogen-bond forces of CQAs with BSA increased the stability of protein structure, and lead to the increases of α-helix contents. Especially, in Tris-HCl buffer, the increases of α-helix contents were the most obvious (8.16, 14.14, and 5.15% changes for 5-CQA, 4-CQA, and 3-CQA, respectively), which coincided with the results of FTIR spectra. Similarly, 4-CQA induced the strongest changes in the α-helical content of BSA in PBS buffer, Tris-HCl buffer and H_2_O, corresponding to an increase of 1.97, 14.14, and 4.84%, respectively. For 5CQA-BSA system, the changes were weak (0.08, 5.35, and 0.69% for PBS buffer, Tris-HCl buffer and H_2_O, respectively). The results demonstrated that CQAs could cause some minor conformational changes on BSA, and BSA became partially ordered due to BSA-CQAs complex formation.

#### Synchronous Fluorescence

**Figure 4:**
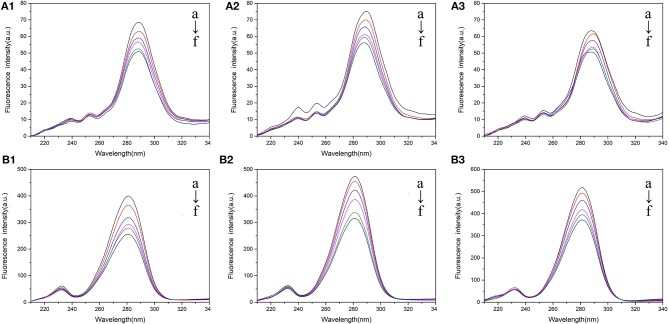
Synchronous fluorescence spectra of BSA mixed with various concentrations of 5-CQA in PBS buffer (1), Tris-HCl buffer (2), and H_2_O (3). **(A)** =15 nm; **(B)** =60 nm. The concentration of 5-CQA is 0, 1, 2, 3, 4, 5 × 10^−6^ M from a to f.

Synchronous fluorescence spectrum was usually employed to study the effect of ligand to protein conformation. When the scanning interval Δλ was set at 15 or 60 nm, the synchronous fluorescence of BSA stands for the characteristic fluorescence of *Tyr* or *Trp*, respectively (Cui et al., 2009). The maxima emission of *Tyr* and *Trp* residues is related to their polarity microenvironment. Figure 4 showed the synchronous fluorescence spectra of BSA with different concentration of 5-CQA, and the synchronous fluorescence spectra of BSA in the presence of 3-CQA and 4-CQA are, respectively shown in Supplementary Figures 6, 7. After addition of CQAs, the fluorescence intensities of *Tyr* and *Trp* were all decreased in three solvent environments. However, the fluorescence intensity, as well as the fluorescence quenching, of *Trp* residues were much higher than that of *Tyr* residues. Therefore, CQAs were closer to *Trp* residue in the interaction process with BSA. Beyond that, there were no changes in the maxima emission of *Trp* residues. However, the maxima emission of *Tyr* showed a slight blue shift with 0.5–2 nm, which showed that the hydrophobicity around *Tyr* was enhancement and the polarity microenvironment reduced.

#### 3D Fluorescence Spectrum

3D fluorescence spectrum has become more and more popular to detect the conformational changes of proteins in recent years. Due to the presence of endogenous fluorescent chromophoric groups, three-dimensional fluorescence spectroscopy could comprehensively and efficiently characterize changes in conformation and spatial structure of proteins. Three-dimensional fluorescence spectra of CQAs binding with BSA were shown in Supplementary Figure 8. There were two peaks in 3D fluorescence spectrum of BSA which were related to structure of proteins. Peak 1 showed the structure information of *Tyr* and *Trp* residues, while Peak 2 was related to the backbone structures of the polypeptide chain (caused by the n-π* transition of the carbonyl group in the protein), and could reveal the secondary structure changes of protein (Paul and Guchhait, 2011).

**Figure 5:**
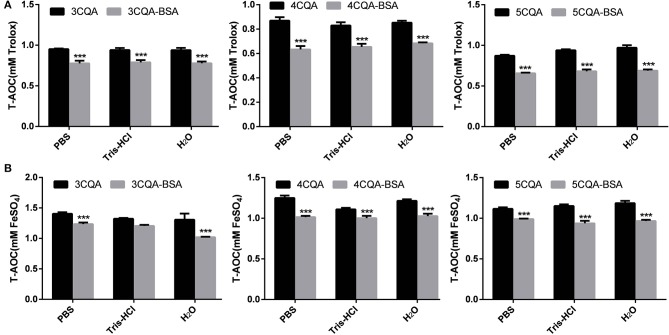
DPPH **(A)** and ABTS **(B)** scavenging activity of CQAs and BSA-CQAs complex in different solvent systems. ***P < 0.001.

As seen from Figure 5, the fluorescence intensities of Peak 1 and Peak 2 were all reduced when addition of CQAs in different solvent environments. Beyond that, related peak positions were all blue shifted, which indicated that the increased-hydrophobicity around *Tyr* and *Trp* residues. The fluorescence quenching percentages of Peak 1 and Peak 2 of CQAs in Tris-HCl buffer were larger than that in PBS buffer and H_2_O. Compared with 5-CQA-BSA complex and 4-CQA-BSA complex, 3-CQA-BSA complex often had smaller fluorescence quenching percentage of Peak 1 and Peak 2. These results not only showed that the binding induced conformational changes of BSA, but also indicated that conformational change were more obvious in Tris-HCl buffer and 3-CQA had the weaker interaction with BSA, which was in accord with the information obtained from CD spectrum.

### Antioxidant Activity Assessment

Due to the presence of phenolic hydroxyl groups, CQAs has remarkable antioxidant properties. The interaction between small molecule and proteins could improve the stability of small molecule. Zhou et al. found that both DPPH and ABTS radical scavenging rates of anthocyanin had been decreased when addition of BSA (Zhou et al., 2013). Zheng et al. found that the stability of bog bilberry anthocyanin extract (BBAE) was also preserved by interacting with BSA, which had less change in the absorbance and antioxidant activity (Zheng et al., 2009). Therefore, contrast experiment of the antioxidant properties of CQAs and CQAs-BSA complex could not only reflect the effect of the binding on the antioxidant activity of the drug, but also display the degree of the interaction between CQAs and BSA. These results were shown in Figure 5. Both results of FRAP and ATBS method showed that the total antioxidant capacity of 5-CQA were stronger than that of 4-CQA and 3-CQA, and there were no significant differences in three solvent systems. However, with the addition of BSA, the total antioxidant capacities of three CQAs were all decreased. The phenolic hydroxyl group of CQAs could react with oxygen radical to form a resonance-stable phenoxy radical, and the oxidative chain reaction stop. The reduction of total antioxidant capacities of CQAs after binding with BSA may be due to the fact that the hydroxyl groups in the CQAs participate in the formation of hydrogen bonds in the BSA interaction, which resulting in the shielding of the hydroxyl groups.

### Identification Degradation Products in BSA Solution

During the course of the study, we found an interesting experimental phenomenon that all the CQAs would degrade into unknown components in BSA solution no matter what dissolved in PBS buffer, Tris-HCl buffer or water. Therefore, HPLC-Q-TOF MS^n^ was used to analyze the degradation products of CQAs in BSA solution. The negative total ion chromatogram (TIC) of BSA solution was shown in Supplementary Figure 9.

Compound 1 (C_7_H_12_O_6_) was provisionally recognized as quinic acid. The [M-H]^−^ ion at *m/z* 191.05650 produced the fragment ion at *m/z* 173 [M-H-H_2_O]^−^, *m/z* 137 [M-H-3H_2_O]^−^, and 93 [M-H-3H_2_O-CO_2_]^−^. Compound 2 (C_9_H_8_O_4_) yielded [M-H]^−^ at *m/z* 179.0350. The quasi-molecular ion yielded the ions at *m/z* 135 [M-H-CO_2_]^−^, 117 [M-H-CO_2_-H_2_O]^−^. The product ion at *m/z* 134 was yielded by losing COOH. The ion also generated minor ions at *m/z* 107 [M-H-COOH-C_2_H_3_]^−^ and 89 [M-H-COOH-C_2_H_3_-H_2_O]^−^. Comparing with reference, compound 2 was affirmatively deduced as caffeic acid. Compound 3 (C_10_H_10_O_4_) yielded [M-H]^−^ at *m/z* 193.0510. The quasi-molecular ion generated the ESI-MS/MS ion at *m/z* 353 by losing CH_3_OH. A series of ions at *m/z* 134 [M-H-COOCH_3_]^−^ and *m/z* 133 [M-2H-COOCH_3_]^−^ were generated. Comparing with reference, compound 3 was affirmatively identified as methyl caffeate.

These results showed that the degradation of CQAs in BSA solution mainly involved the hydrolysis of ester bond and the methyl esterification of carboxyl group. The specific degradation process was shown in Supplementary Scheme 1. Many research showed that CGA could be absorbed in the small intestine and part of them were hydrolysed into caffeic acid, ferulic acid, gallic acids, and quininic acid under the effect of gut microbiota (Lafay et al., 2006; Ohnishi et al., 2006; Duda-Chodak, 2012). Wu et al. investigated the effects of CGA and five metabolites (caffeic, quininic, ferulic, gallic, and vanillic acids) on cholesterol efflux. CGA and its metabolites potently reduced atherosclerosis development in ApoE(-/-) mice and these five metabolites might be the potential active compounds accounting for the *in vivo* effect of CGA (Wu et al., 2014). Therefore, the degradation of CQAs in BSA could be similar to the metabolic process in small intestine.

### Molecular Docking Studies

**Figure 6:**
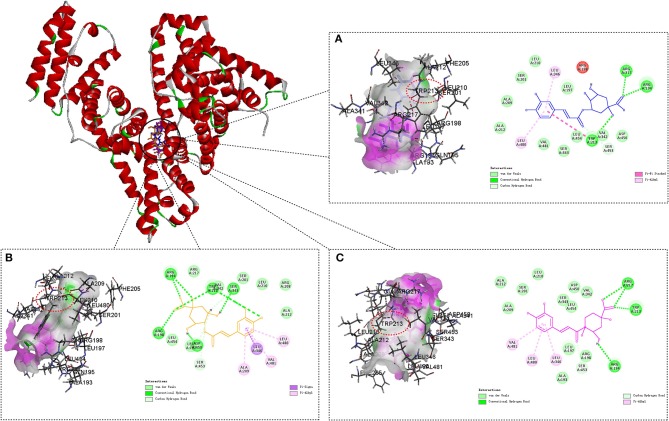
Molecule docking results for the interaction of 3-CQA **(A)**, 4-CQA **(B)**, and 5-CQA **(C)** with BSA. Compound docked in the binding pocket of BSA are placed at the left of the dotted box, and two-dimensional schematic representation of the interaction are shown at the right of the dotted box. Hydrogen bond depicts in green dashed line.

The Discovery Studio programs were employed to investigate the interaction of CQAs and BSA, the most possible binding mode is shown in Figure 6. Corresponding calculated results and key amino acids in active pocket interaction with ligand are shown in Supplementary Table 4.

It could be clearly shown that all the three CQAs binding within the Sudlow's site I in subdomain IIA of BSA, which conformed to our findings in competitive binding experiments. It was worth noting that the tryptophan residue (*Trp* 213) of BSA binding with CQAs by hydrogen bonds, which could provide an evidence to elucidate the fluorescence quenching of BSA binding with CQAs. The results showed that the binding forces of CQAs with BSA were hydrogen-bond interaction and Van der Waals force, which was consistent with our experimental results. In addition, the aromatic nucleus of CQAs could bind with a few hydrophobic residues of BSA by Pi-Alkyl, like *Leu* 346 and *Leu* 480 for 3-CQA, *Ala* 209, *Leu* 480, and *Val* 481 for 4-CQA, *Leu* 346, *Leu* 480, and *Val* 481 for 5-CQA. That could increase the stability of phenolic hydroxyl groups and might be the main reason of the total antioxidant capacities of three CQAs were all decreased with the addition of BSA.

The LibDock score of the degradation products (quinic acid, caffeic acid and methyl caffeate) were all less than NPS (original ligand) (Bujacz et al., 2014), indicating that they could weakly or not bind with site I. Li et al. also found that the binding constant of chlorogenic acid was higher than that of caffeic acid, which suggested that the presence of ester bond increased the affinities (Li et al., 2010). Therefore, combined with the results of HPLC-MS/MS, we could make the following assumptions: (1) The BSA solution could have two kinds of enzymes—hydrolase and esterifying enzyme. Hydrolase could hydrolyse the ester bonds in CQAs to release caffeic acid and quinic acid. Traces of caffeic acid and methyl alcohol could react to methyl caffeate by esterifying enzyme. (2) The BSA binding affinity of three CQAs was strong, but the interaction between their degradation products with BSA was weak. (3) The binding affinity changes of CQAs interacted with BSA may relate to degradation degree of CQAs. The order of hydrolase activity in different solvent environments may be PBS>H_2_O>Tris-HCl, and the degradation of CQAs in Tris-HCl buffer could be poorer than that in PBS buffer or H_2_O, which lead to less degradation products and higher binding affinity.

## Discussion and Conclusion

This study investigated the binding mechanism of CQAs and BSA in PBS buffer, Tris-HCl buffer and H_2_O by multi-spectroscopic method, antioxidant activity assessment and docking. The results indicated that the intrinsic fluorescence of BSA was quenched by CQAs and the interaction was static quenching with the formation of a non-fluorescent complex. The binding of CQAs and BSA was spontaneous, and Van der Waals forces and hydrogen-bond interaction occupied crucial roles in the binding. The solvent environments and the structure of CQAs had a significant impact on the interaction. All the three CQAs could bind to Site I in Domain IIA. The binding constants, binding distances, free energy changes, FTIR spectrum, synchronous and 3D fluorescence indicated that the binding affinity of CQAs had a descending order of Tris-HCl > H_2_O > PBS. These results also indicated that 3-CQA had the weakest interaction with BSA, which may due to its larger polarity. CD spectra results showed CQAs could influence the secondary structures of BSA by decreasing the α-helix percentage.

Beyond that, the interaction of BSA and CQAs could reduce the total antioxidant capacities of CQAs, and different solvents had no significant effect on it. The main reason could be the aromatic nucleus of CQAs could bind with a few hydrophobic residues of BSA by Pi-Alkyl, and this non-covalent bond could increase the stability of phenolic hydroxyl groups. The first and second dissociation constant (p*K*a) of CQAs in water system is 3.4–3.6 and 8.45, respectively. This study was carried out under the pH 7.4. Therefore, if this hypothesis was right and the binding with BSA could shield the hydroxyl groups, p*K*a of CQAs may increase and the dissociation of CQAs may decrease at pH 7.4. Therefore, more research on the effect of binding with BSA on the p*K*a value of CQAs is recommended in the future.

Lafay et al. fed rats diets supplemented with chlorogenic acids to determine the form of absorption through the intestinal mucosa and gastrointestinal absorption sites. They found that most of chlorogenic acids were absorbed into the stomach and small intestine in its intact form (Lafay et al., 2006). In this study, the presence of esterase in BSA solution resulted in the hydrolysis of CQAs, which might have influence on the accuracy of the determined results and cannot reflect the drug-protein binding *in vivo* well. Therefore, the binding parameters determined in Tris-HCl buffer may be more meaningful due to poor degradation degree of CQAs. Our study also reminded that for the low stability compounds in BSA solution, plasma protein binding measurements should be investigated in different buffers to evaluate drug-BSA binding affinity comprehensively and objectively.

In all, this study provided a complete evaluation of interaction profiles between CQAs and BSA, and made a sufficient contribution to monitoring the binding affinities changes which were influenced by solvent types and the structure of CQAs.

## Data Availability Statement

The raw data supporting the conclusions of this manuscript will be made available by the authors, without undue reservation, to any qualified researcher.

## Author Contributions

BL, WZ, and QJ designed the experiments and wrote the paper. QJ and XC collected and analyzed the data. YJ, LJ, and QJ prepared reagents, materials, and analysis tools.

### Conflict of Interest

The authors declare that the research was conducted in the absence of any commercial or financial relationships that could be construed as a potential conflict of interest.
