# Supplementary material for: Study on the Interactions Between Caffeoylquinic Acids With Bovine Serum Albumin: Spectroscopy, Antioxidant Activity, LC-MSn, and Molecular Docking Approach
Source: Front Chem. 2019 Dec 6;7:840. doi: 10.3389/fchem.2019.00840 (PMC6909939; doi:10.3389/fchem.2019.00840)
Supplement: Supplementary file 1 [file Table_1.docx]

Supplementary Material

# Supplementary Figures and Tables

## Supplementary Figures

**
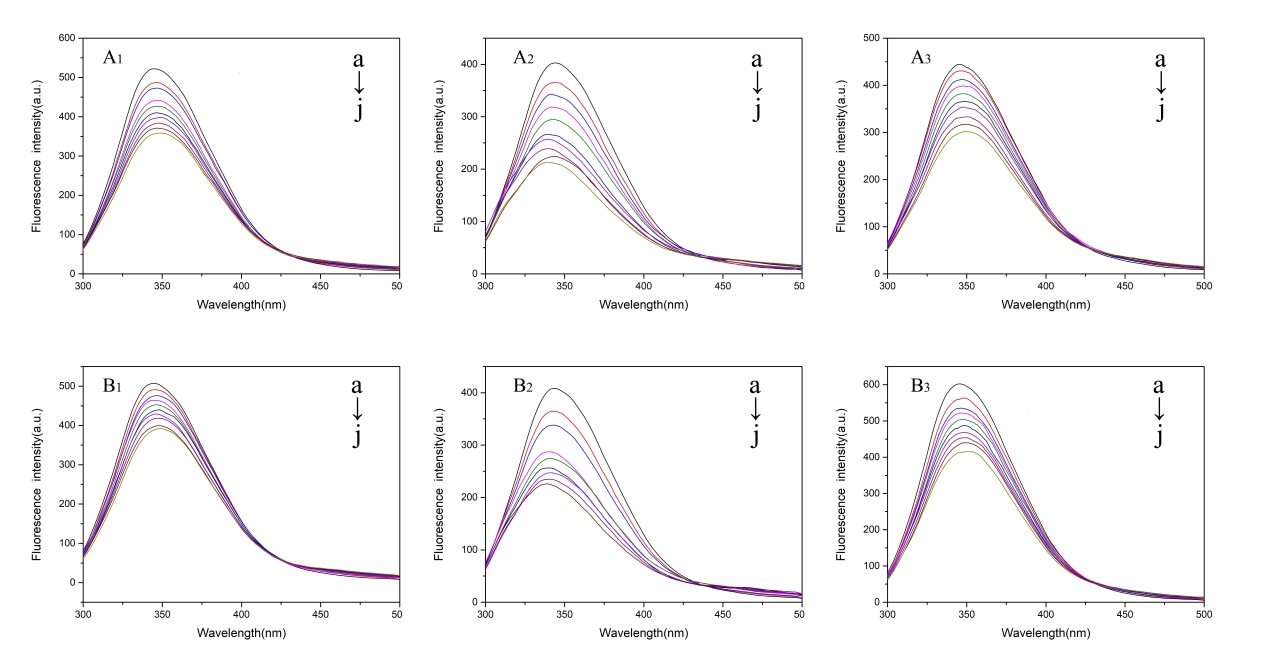
**

**Supplementary Figure 1.** The fluorescence spectra of 2.0 × 10-7 M BSA mixed with various concentrations of 3-CQA (A) and 4-CQA (B) at 300K in PBS buffer (1), Tris-HCl buffer (2) and H2O (3). The concentration of 5CQA is 0.0, 1.0, 1.5, 2.0, 2.5, 3.0, 3.5, 4.0, 4.5, 5.0 × 10^-6^ M from a to j.

**
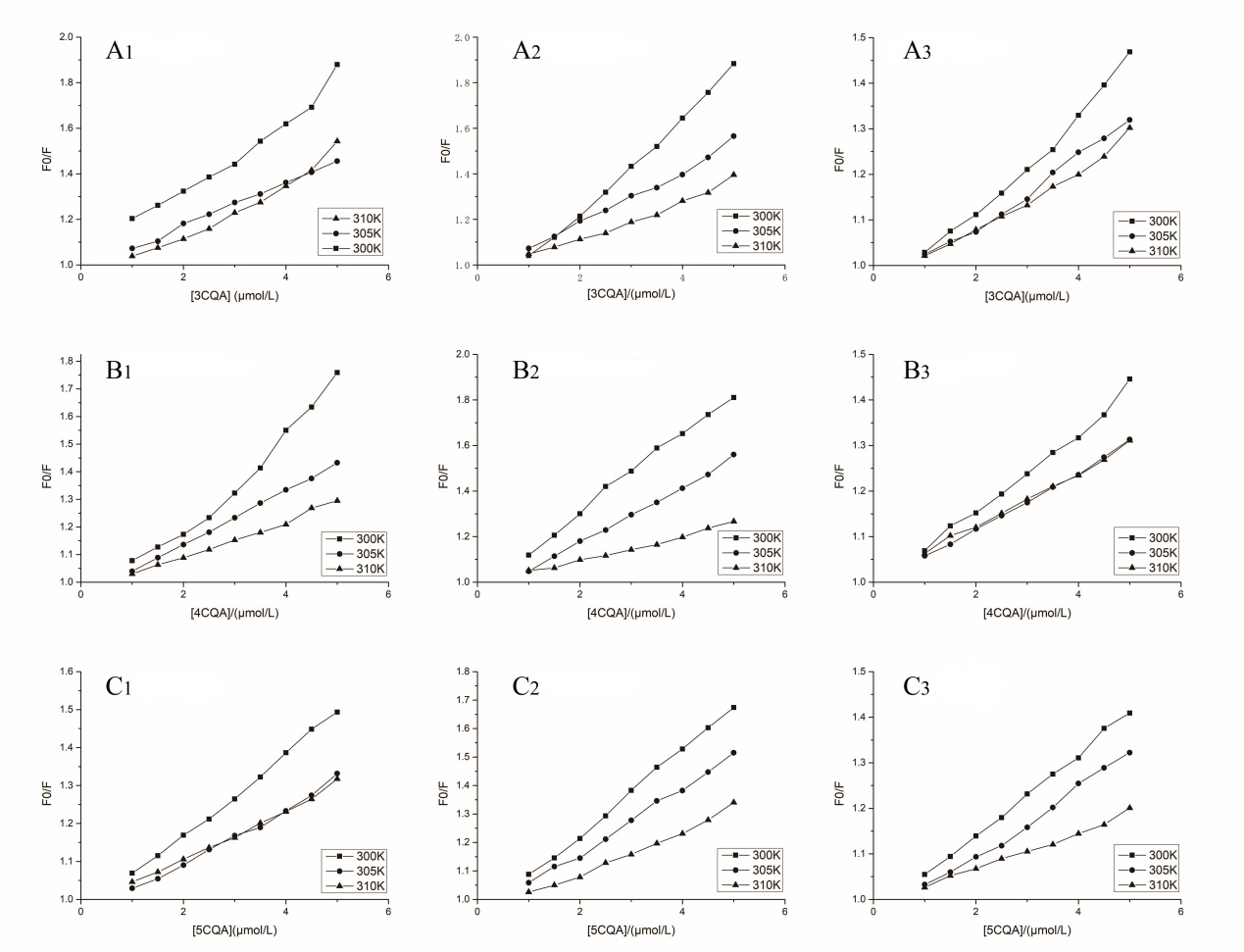
**

**Supplementary Figure 2.** SterneVolmer curves of BSA fluorescence quenched by 5-CQA (A), 4-CQA (B) and 3-CQA (C) in PBS buffer (1), Tris-HCl buffer (2) and H_2_O (3) at different temperature.

**
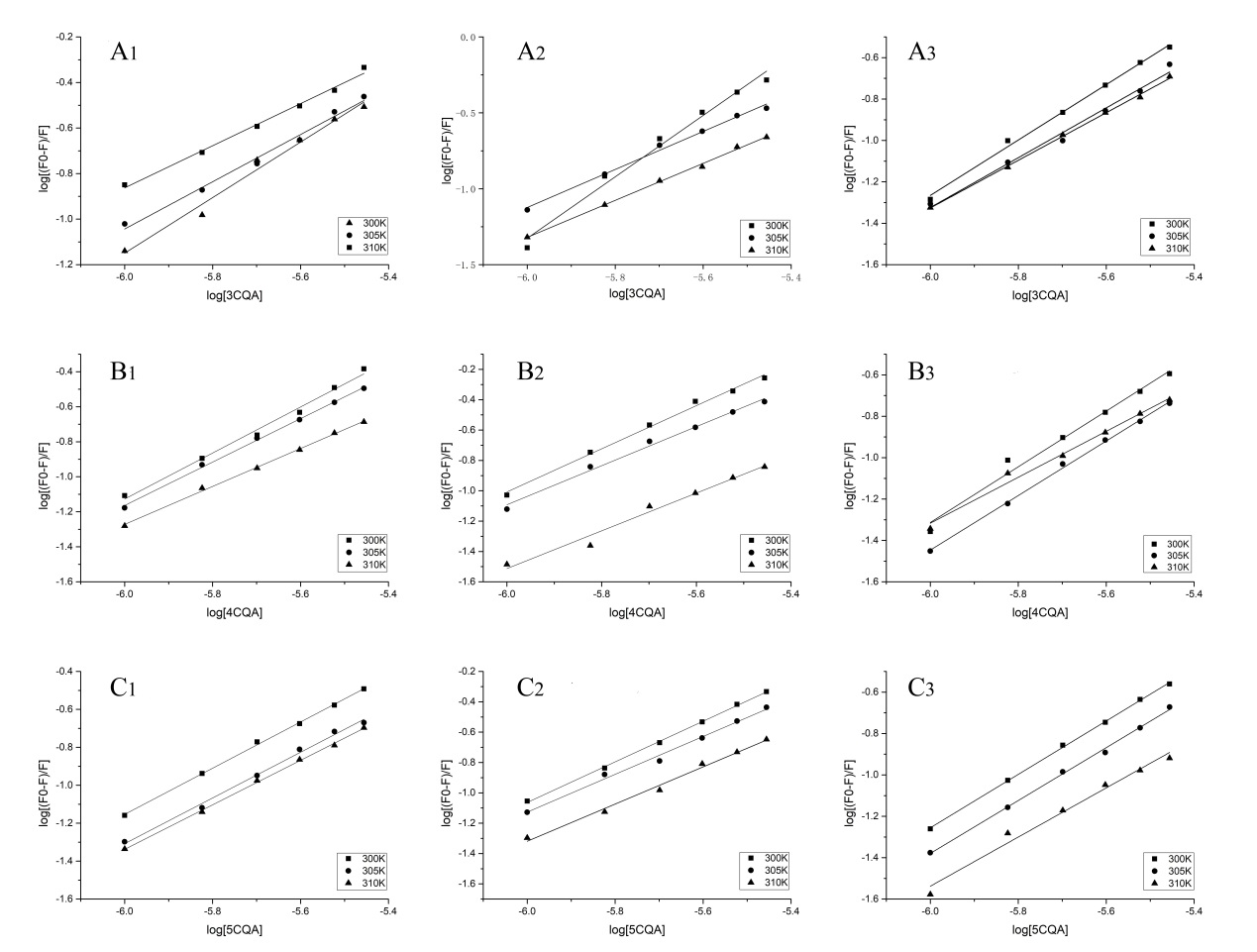
**

**Supplementary Figure 3.** Double-log plots of BSA fluorescence quenched by 5-CQA (A), 4-CQA (B) and 3-CQA (C) in PBS buffer (1), Tris-HCl buffer (2) and H_2_O (3) at different temperature.


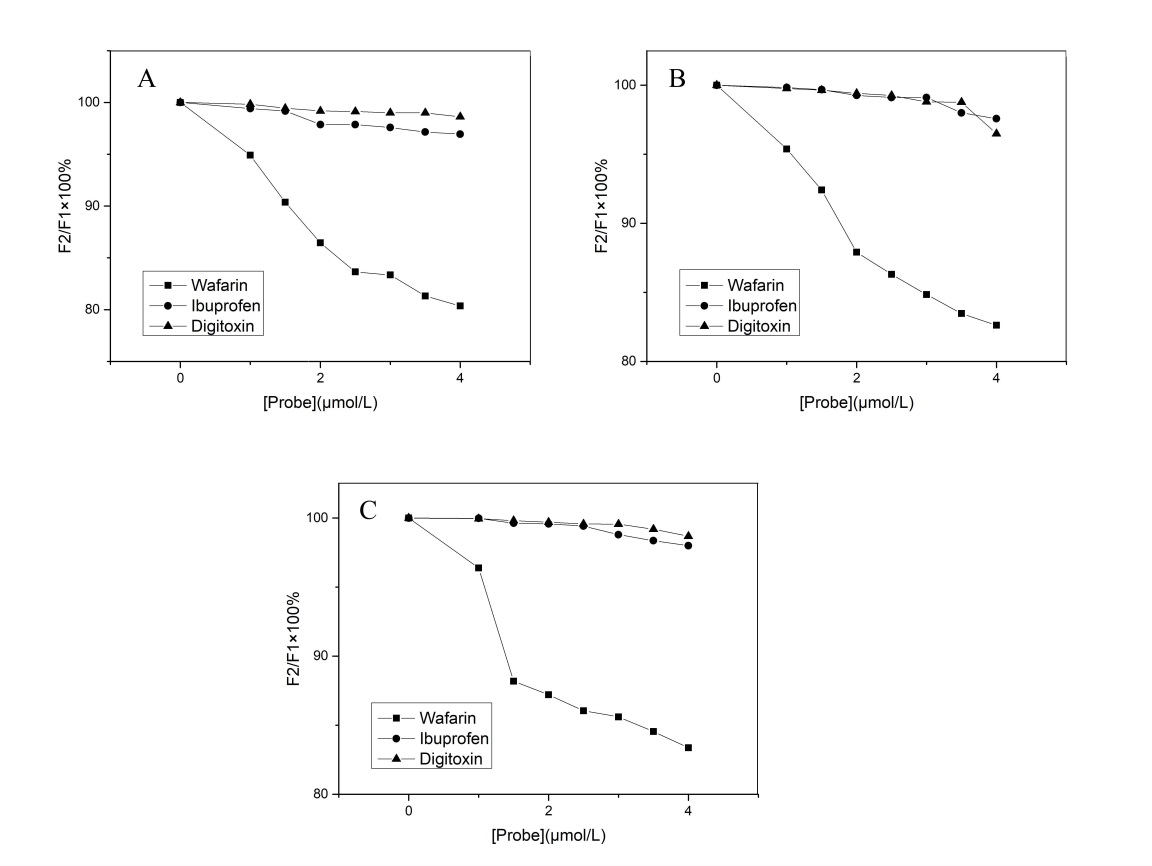


**Supplementary Figure 4.** The probe displacement percentage of BSA mixed with various concentrations of 5-CQA (A), 4-CQA (B) and 3-CQA (C).

**
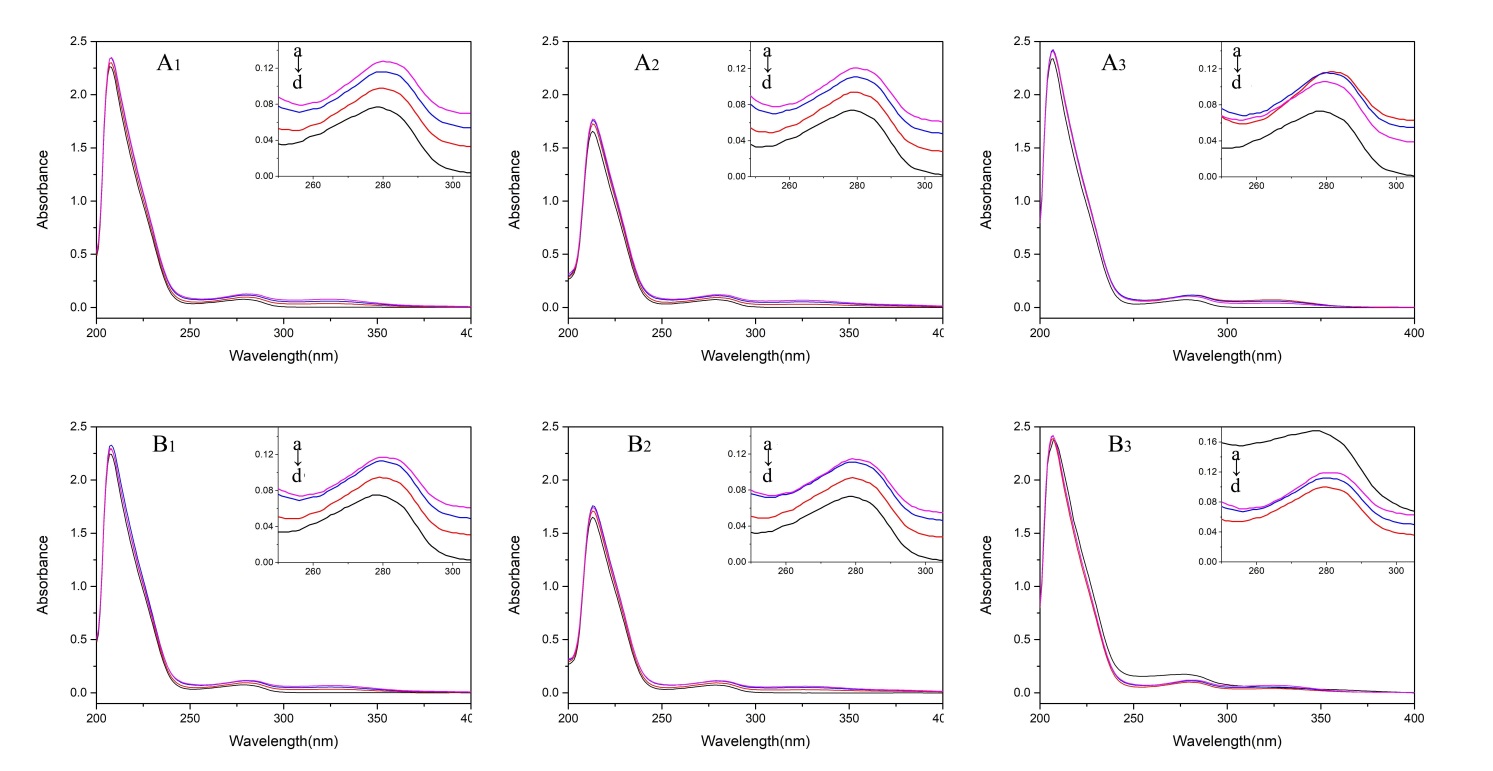
**

**Supplementary Figure** **5**. UV-vis absorption spectra of 2.0 × 10^-7^ M BSA in the presence of 3-CQA (A) and 4-CQA (B) in PBS buffer (A), Tris-HCl buffer (B) and H_2_O (C). The concentration of 3-CQA and 4-CQA is 0, 2, 4, 6 × 10^-6^ M from a to d.

**
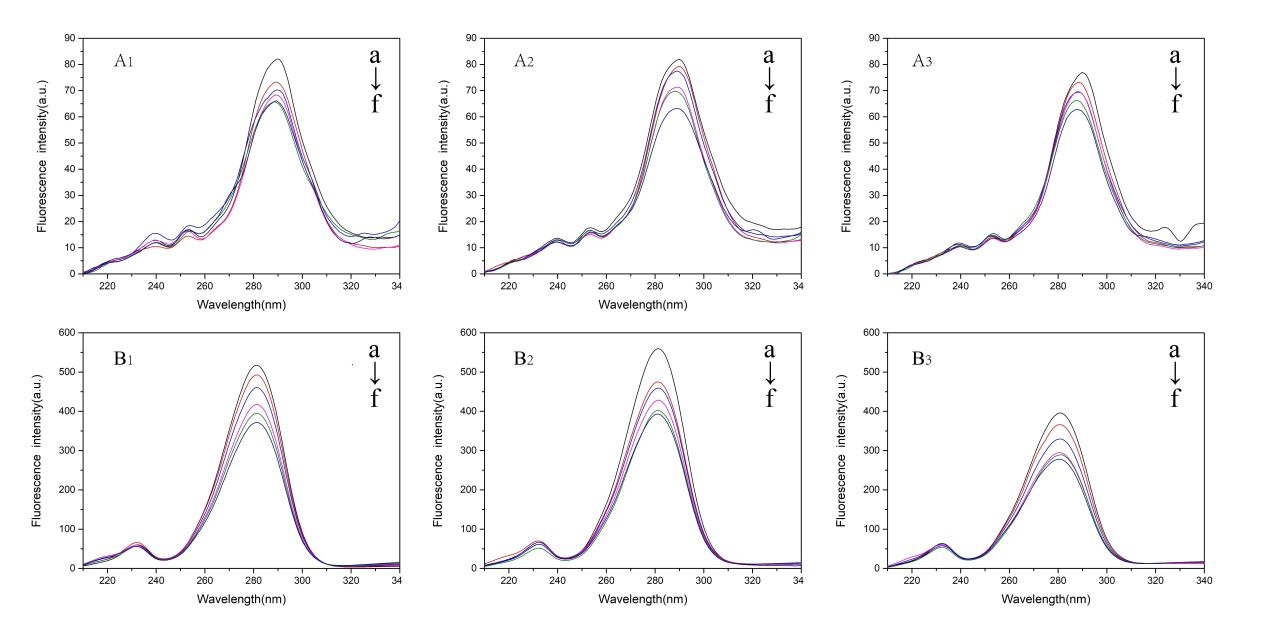
**

**Supplementary Figure** **6.** Synchronous fluorescence spectra of BSA mixed with various concentrations of 3-CQA in PBS buffer (1), Tris-HCl buffer (2) and H_2_O (3). (A) Δ*λ*=15 nm; (B) Δ*λ*=60 nm. The concentration of 3-CQA is 0, 1, 2, 3, 4, 5 × 10^-6^ M from a to f.

**
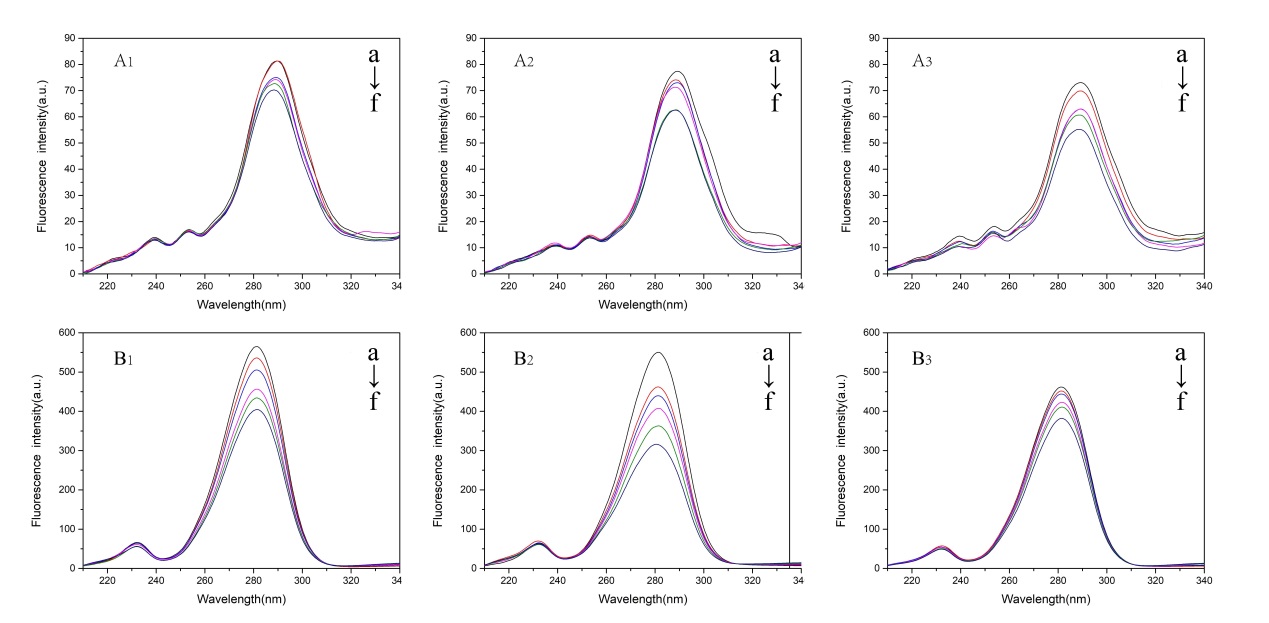
**

**Supplementary Figure** **7.** Synchronous fluorescence spectra of BSA mixed with various concentrations of 4-CQA in PBS buffer (1), Tris-HCl buffer (2) and H_2_O (3). (A) Δ*λ*=15 nm; (B) Δ*λ*=60 nm. The concentration of 3-CQA is 0, 1, 2, 3, 4, 5 × 10^-6^ M from a to f.

**
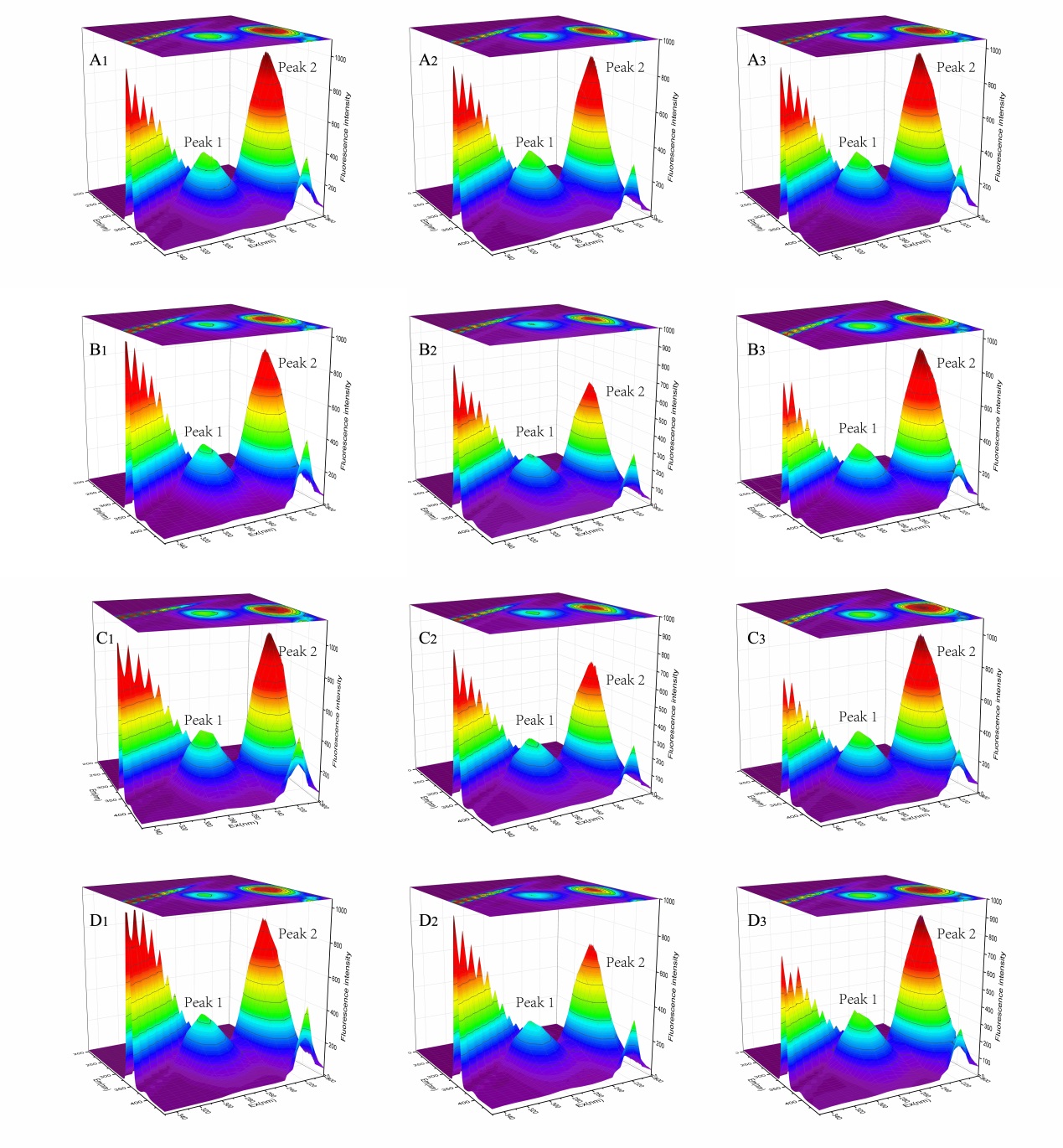
**

**Supplementary Figure** **8.** Three-dimensional fluorescence spectra of BSA (A), 3-CQA-BSA (B) and 4-CQA-BSA (C) and 5-CQA-BSA (D) complex in (1) PBS buffer, (2) Tris-HCl buffer and (3) H_2_O.


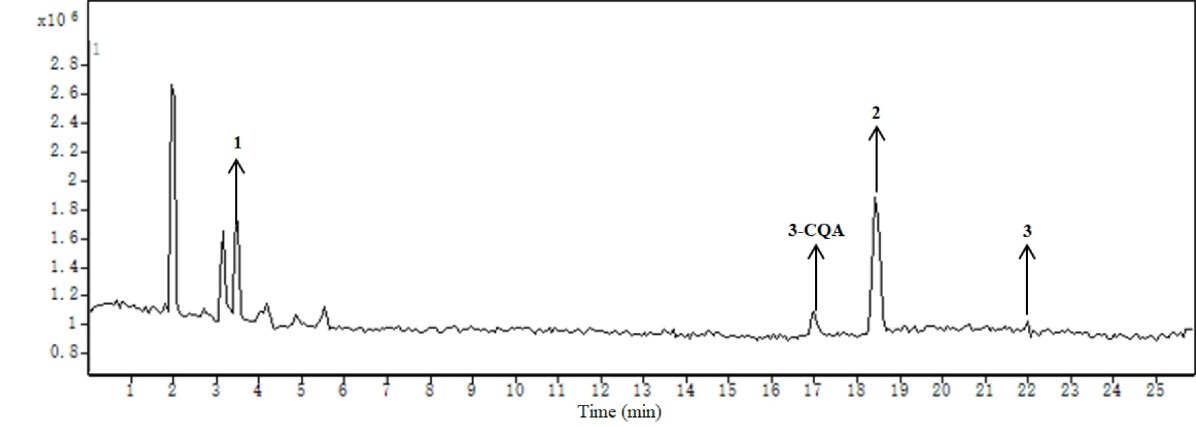


**Supplementary Figure** **9.** TIC chromatogram of BSA solution in negative ion mode.





**Supplementary Scheme 1.** Degradation of 3-CQA/4-CQA/5-CQA in BSA solution. (a) hydrolyzation; (b) methyl esterification.

## Supplementary Tables

**Supplementary Table 1** The influences of CQAs to the fluorescence spectra of BSA

| Compound | Solution | *λ*a-*λ*j（nm） | Fluorescence quenching rate（%） |
| --- | --- | --- | --- |
| 5-CQA | PBS | -2 | 35.34 |
|  | H_2_O | -3 | 32.09 |
|  | Tris-HCl | 4.5 | 47.07 |
| 4-CQA | PBS | -4 | 22.65 |
|  | H_2_O | -5.5 | 30.99 |
|  | Tris-HCl | 3.5 | 35.83 |
| 3-CQA | PBS | -4.5 | 32.93 |
|  | H_2_O | -4 | 28.97 |
|  | Tris-HCl | 1.5 | 40.14 |

**Supplementary Table** **2** Binding and thermodynamic parameters for the interaction between BSA and 3-CQA at different temperatures

| Solution | T (K) | K_SV_  (L·mol^-1^) | Ka  (L·mol^-1^) | n | Δ*H*  (kJ·mol^-1^) | Δ*S*  (J·mol^-1^·k^-1^) | Δ*G*  (kJ·mol^-1^) |
| --- | --- | --- | --- | --- | --- | --- | --- |
| PBS | 300 | 10.03×10^4^ | 1.41×10^6^ | 1.2174 | -80.41 | -150.33 | -35.31 |
|  | 305 | 6.75×10^4^ | 8.32×10^5^ | 1.2047 |  |  | -34.56 |
|  | 310 | 6.15×10^4^ | 5.01×10^5^ | 1.1729 |  |  | -33.81 |
| Tris-HCl | 300 | 15.26×10^4^ | 9.27×10^6^ | 1.3385 | -166.51 | -422.58 | -39.74 |
|  | 305 | 11.39×10^4^ | 2.24×10^6^ | 1.2462 |  |  | -37.62 |
|  | 310 | 7.02×10^4^ | 1.08×10^6^ | 1.2252 |  |  | -35.51 |
| H_2_O | 300 | 8.88×10^4^ | 3.08×10^6^ | 1.2907 | -159.56 | -406.07 | -37.74 |
|  | 305 | 6.66×10^4^ | 1.93×10^6^ | 1.2773 |  |  | -35.71 |
|  | 310 | 3.72×10^4^ | 3.87×10^5^ | 1.1875 |  |  | -33.68 |

**Supplementary Table** **3** Binding and thermodynamic parameters for the interaction between BSA and 4-CQA at different temperatures

| Solution | T (K) | K_SV_  (L·mol^-1^) | Ka  (L·mol^-1^) | n | Δ*H*  (kJ·mol^-1^) | Δ*S*  (J·mol^-1^·k^-1^) | Δ*G*  (kJ·mol^-1^) |
| --- | --- | --- | --- | --- | --- | --- | --- |
| PBS | 300 | 13.29×10^4^ | 5.70×10^6^ | 1.3138 | -271.59 | -774.09 | -39.36 |
|  | 305 | 9.80×10^4^ | 1.88×10^6^ | 1.2398 |  |  | -35.49 |
|  | 310 | 6.00×10^4^ | 1.69×10^5^ | 1.0835 |  |  | -31.62 |
| Tris-HCl | 300 | 18.94×10^4^ | 3.32×10^7^ | 1.4214 | -217.90 | -588.56 | -41.33 |
|  | 305 | 12.04×10^4^ | 4.21×10^6^ | 1.2871 |  |  | -38.40 |
|  | 310 | 4.71×10^4^ | 8.87×10^5^ | 1.2434 |  |  | -35.45 |
| H_2_O | 300 | 8.36×10^4^ | 5.78×10^6^ | 1.3458 | -251.26 | -705.46 | -39.62 |
|  | 305 | 6.05×10^4^ | 2.88×10^6^ | 1.3177 |  |  | -36.09 |
|  | 310 | 5.77×10^4^ | 2.21×10^5^ | 1.1102 |  |  | -32.57 |

**Supplementary Table** **4** Molecule docking results for the interaction of CQAs and degradation product with BSA.

| Compound | Binding forces | Score | Key amino acids |
| --- | --- | --- | --- |
| 5-CQA | a, b^*^ | 137.03 | *Trp* 213, *Arg* 217, *Arg* 194 |
| 4-CQA | a, b^*^ | 142.32 | *Trp* 213, *Arg* 198, *Arg* 194, *Asp* 450,  *Ser* 343 |
| 3-CQA | a, b^*^ | 135.17 | *Trp* 213, *Arg* 217, *Arg* 194 |
| caffeic acid |  | 86.77 |  |
| methyl caffeate |  | 88.38 |  |
| quinic acid |  | 82.98 |  |
| NPS (original ligand) |  | 104.42 |  |

*: a is hydrogen bonds; b is van der Waals forces.
